# The Role of Additives in Warm Mix Asphalt Technology: An Insight into Their Mechanisms of Improving an Emerging Technology

**DOI:** 10.3390/nano10061202

**Published:** 2020-06-19

**Authors:** Paolino Caputo, Abraham A. Abe, Valeria Loise, Michele Porto, Pietro Calandra, Ruggero Angelico, Cesare Oliviero Rossi

**Affiliations:** 1 Department of Chemistry and Chemical Technologies, University of Calabria, 87036 Arcavacata di Rende (CS), Italy; paolino.caputo@unical.it (P.C.); abraham.abe@unical.it (A.A.A.); valeria.loise@unical.it (V.L.); michele.porto@unical.it (M.P.); 2 CNR-ISMN, National Research Council, Institute for the Study of Nanostructured Materials, Via Salaria km 29.300, 00015 Monterotondo Stazione (RM), Italy; pietro.calandra@cnr.it; 3 Department of Agricultural, Environmental and Food Sciences (DIAAA), University of Molise, Via De Sanctis, 86100 Campobasso (CB), Italy

**Keywords:** warm mix asphalt, binder, surface free energy, wax, emulsifiers, surfactants, zeolites, contact angle, bitumen, aggregates, WMA, viscosity

## Abstract

The asphalt industry’s incentive to reduce greenhouse gas emissions has increased since the 1990s due to growing concerns on environmental issues such as global warming and carbon footprint. This has stimulated the introduction of Warm Mix Asphalt (WMA) and its technologies which serve the purpose of reducing greenhouse gas emissions by reducing the mixing and compaction temperatures of asphalt mix. WMA gained popularity due to the environmental benefit it offers without compromising the properties, performance and quality of the asphalt mix. WMA is produced at significantly lower temperatures (slightly above 100 °C) and thus results in less energy consumption, fewer emissions, reduced ageing, lower mixing and compaction temperatures, cool weather paving and better workability of the mix. The latter of these benefits is attributed to the incorporation of additives into WMA. These additives can also confer even better performance of WMA in comparison to conventional Hot Mix Asphalt (HMA) methods. Even though there are recommended dosages of several WMA additives, there is no general standardized mixture design procedure and this makes it challenging to characterize the mechanism(s) of action of these additives in the warm mix. The effects of the addition of additives into WMA are known to a reasonable extent but not so much is known about the underlying interactions and phenomena which bring about the mechanism(s) by which these additives confer beneficial features into the warm mix. Additives in a certain way are being used to bridge the gap and minimize or even nullify the effect of the mixing temperature deficit involved in WMA processes while improving the general properties of the mix. This review presents WMA technologies such as wax, chemical additives and foaming processes and the mechanisms by which they function to confer desired characteristics and improve the durability of the mix. Hybrid techniques are also briefly mentioned in this paper in addition to a detailed description of the specific modes of action of popular WMA technologies such as Sasobit, Evotherm and Advera. This paper highlights the environmental and technical advantages of WMA over the conventional HMA methods and also comprehensively analyzes the mechanism(s) of action of additives in conferring desirable characteristics on WMA, which ultimately improves its durability.

## 1. Introduction

**Figure 1:**
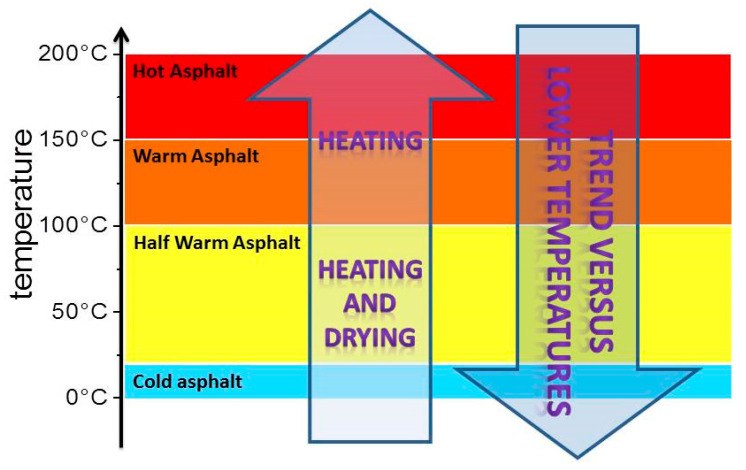
Classification by temperature range.

Since the 1900s, Hot Mix Asphalt (HMA) has been the most common and generally accepted technology for asphalt pavement construction. Road pavements are made up of over 93% stones with less than 7% of bitumen functioning as a binder. Bitumen is a soft material made up of several components and is a by-product of petroleum industry processes. Its chemical composition is complex and it can be characterized using special methods [1]. In order to ensure proper coating of aggregates and provide sufficient workability of HMA, both asphalt binder and aggregates are heated up to high temperatures between 150 °C and 180 °C. This results in high energy costs and the emission of several greenhouse gases (mainly CO_2_). The Kyoto Treaty was developed as a result in 1997, thus setting the objective for European countries to develop policies and technologies in order to meet greenhouse gas reduction requirements [2]. Warm Mix Asphalt (WMA) technologies were developed to produce asphalt at temperatures slightly above 100 °C, with performances and characteristics equivalent to or even sometimes better than that of conventional HMA. WMA technologies mostly focus on the binder (bitumen) by adding different additives to improve its properties [3,4,5,6,7]. These technologies, which produce asphalt between 110 °C and 140 °C, facilitate proper coating of the aggregates and hence the workability and compactibility of the mix while also reducing production and compaction temperatures by 20–40 °C. This reduces energy consumption, minimizes fume and odor emissions and also creates a cooler working environment for asphalt workers [8]. Figure 1 shows the position of WMA among the different techniques ranging from cold to hot mixes.

Several techniques exist for the production of WMA. The three generally most accepted are those using (i) *organic additives*; (ii) *chemical additives*; and, (iii) *foaming techniques* [9]. Organic additives are usually waxes and fatty amides, which are able to reduce the viscosity of the binder above the melting point of the binder. Common waxes used in the production of WMA are Sasobit^®^ and Asphaltan B^®^. On the other hand, chemical additives are usually emulsifiers and surfactants that do not reduce the binder viscosity but improve the coating of aggregates by reducing the surface energy of the aggregate/binder interface and/or the inner friction. Products such as Rediset^®^ and Evotherm^®^ are often used [10]. Foaming techniques function by reducing the viscosity of the binder, just like organic additive techniques, but only for a short period of time. This is achieved by introducing small amounts of water in the hot binder (bitumen), causing expansion of the bitumen and the formation of a large quantity of foam. Foaming techniques are subcategorized into (i) *water-based processes*, which entail the use of injection foaming nozzles; and, (ii) *water-bearing additives*, which involve the use of minerals in the form of zeolites [8,11].

Aside from the aforementioned WMA techniques, there are also several combined technologies and products used to produce WMA, like zeolites or fibers with organic additives or pallets with fibers. These are called *hybrid techniques*. Hybrid techniques involve a combination of two or more technologies and are used less often. Examples of hybrid techniques are Low Energy Asphalt (LEA) and the Tri-Mix Warm Mix Injection system, which are both technologies that combine chemical and water-based techniques to achieve the required results [2,12,13]. In addition to all of the commonly known techniques for WMA production, an uncommon technique that uses emulsions to pre-coat aggregates is sometimes used. This technique involves the use of stabilized bituminous emulsion to pre-coat aggregates before the main mixing procedure with asphalt binder. Due to the absence of hazardous additives in this specialized emulsion technique, it is designed to produce a more environmentally friendly and cost-effective procedure [14].

Just like any new technology, WMA needs a lot of further research and study. A few concerns like finding a mix design strategy and the need for a comprehensive specification have been identified thus far. Previous research [15,16] confirms that the lack of standardized mixture design procedure of WMA makes it important to identify the mechanism(s) of action of additives. The wide range of mechanisms through which several WMA additives act also provides a wide range of WMA additive product options when deciding how to formulate warm mixes. In addition, since the set of instrumentation, machinery and apparatus available for WMA production contribute to determine the technique to be used, a good knowledge of how and why the additives work gives a realistic evaluation of methods and techniques to be used for future WMA technology. Table 1 shows the range of additives used in warm mix technologies.

Substantial information is known about the effects of additives on warm mix, but the precise mechanism of how these additives work to confer the desired characteristics on WMA is still unclear. The main goal of this paper is to review the most relevant works appearing in the literature on the subject to highlight the mechanism(s) of action of additives and relate them to the effects observed in the WMA technology.

## 2. Organic Additives

Organic WMA additives are known to reduce the bitumen viscosity in order to improve workability. These organic additives are mainly waxes (natural or synthetic) and fatty amides, and they are added to warm mix to lower the viscosity of the asphalt binder at temperatures slightly above 90 °C and to improve lubrication. The reduction in viscosity translates to an increase in stiffness by solidifying into microscopic particles which are distributed uniformly in the mix when it cools [9]. The underlying concept is the fact that waxes suitable for this technique have melting points below the conventional HMA production temperatures, hence they become dispersible in the mix during the WMA production process [11]. They are expected to feed the maltene phase due to their apolar nature. In doing so, their effect would be to better disperse asphaltene clusters at their various levels of aggregation, thus reducing viscosity [17]. At high temperatures, the viscosity of the asphalt binder is an important property because it directly reflects the ability of the binder to be pumped through an asphalt plant, to accurately coat the aggregate in the asphalt concrete mixture and to be compacted to form a new pavement surface [18].

An important fact to consider is that the type of additive must be carefully selected so that its melting point is higher than the expected Warm Mix in-service temperatures. This is done in order to prevent deformation and embrittlement of the asphalt at low temperatures [8].

Paraffin waxes are the ideal and most commonly used additives for enhancing binder flow and quality. Paraffin waxes are of two types: (i) naturally occurring bituminous waxes and (ii) synthetic waxes. Wax manufacturers emphasize the difference between naturally occurring bituminous wax and synthetic wax in terms of their physical properties and structure. The differences are due to the longer carbon chain lengths and the finer crystalline structures of industrially synthesized waxes. An example of an industrially synthesized wax which is known to be very effective in WMA is Sasobit, which has a hydrocarbon chain length in the range of 40 to 115 carbon atoms. Naturally occurring bituminous wax has a hydrocarbon chain length of about 22 to 45 carbon atoms [19].

### 2.1. Sasobit

**Figure 2:**
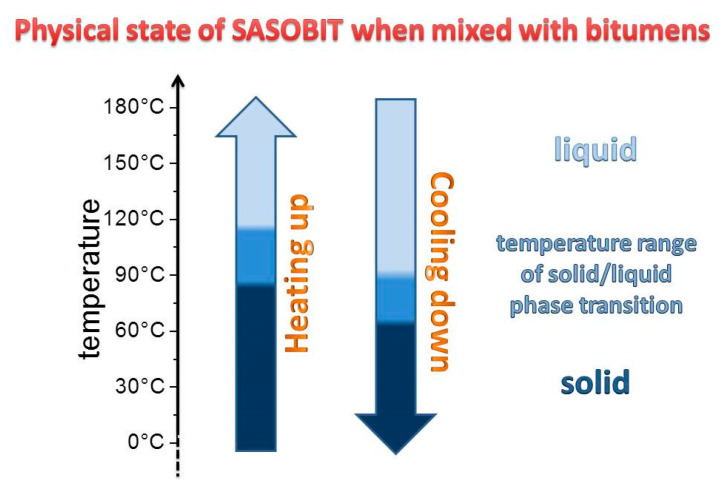
Phase transition mechanism of Sasobit in bitumen binder.

Sasobit is a fine crystalline, long chain aliphatic hydrocarbon produced from coal gasification using the Fischer-Tropsch (FT) process and is otherwise known as an FT paraffin wax. It is generally added in a 3% to 4% proportion with respect to the total asphalt weight. Several studies have reported an increase in the resistance to permanent deformation of WMA mixtures produced with asphalt modified with Sasobit [20,21]. Sasobit acts as a flow modifier in the mix which facilitates the aggregates free movement and coating by the asphalt binder. As Sasobit melts over a temperature range between 85 and 115 °C, it is more dispersible in asphalt binder at temperatures above 115 °C and thus lowers the viscosity of the binder at mixing temperatures [11]. During the cooling process (see Figure 2), Sasobit starts to crystallize at approximately 90 °C and forms a microscopic crystal lattice structure in the bitumen which confers the stiffening effect, and this is responsible for the deformation resistance of the so-modified bitumen [22]. Recent speculation about this phenomenon is that waxes having long hydrocarbon chains (like Sasobit) combine with the binder (bitumen) and alter the hydrocarbon chain length of the binder, thus altering the physical properties of the binder, like stiffness and viscosity [13].

Another contributing factor to the stiffness of the binder is the interaction of long hydrocarbon chains of the waxes with the N-Alkane-rich crystallizing material in asphalt mix [23,24,25]. The small crystalline structure of the Fischer-Tropsch wax molecules reduces brittleness of the paved asphalt road at low temperatures due to the formation of a lattice structure of microscopic particles in the modified binder. Another important feature of the asphalt binder that changes due to the addition of the wax is the number and size of air voids present in the mix. The number and size of air voids is reduced due to the improved flow of the modified bitumen during mixing and compaction procedures. Less air voids result in a greater resistance to ruts induced by traffic demands on the paved asphalt [26].

Several modifications of Sasobit technology profit from its formation of lattice structures in the binder and combine this feature with the introduction of polymers into the wax to achieve target specifications. An example of this is the creation of an additive called Sasoflex which is a compound of a plastomer (Sasobit) combined with an elastomer (styrene-butadiene-styrene) by using a proprietary chemical cross-linking agent (sasolink). The plastomer (Sasobit) fraction reduces the viscosity of the mix at paving temperatures and stiffens the binder at in-service pavement temperatures while the elastomer (SBS) fraction maintains the flexibility at low temperatures [26,27]. Sasobit REDUX is another modification of Sasobit technology, which consists of Fischer-Tropsch synthetic wax (Sasobit) and other petroleum-based waxes. This product has a congealing point of between 72 and 83 °C, thus making it softer than Sasobit. Sasobit REDUX functions using the same lattice-forming mechanism but effectively reducing production and compaction temperatures due to its lower melting point [28].

### 2.2. Licomont 100

Other organic additives like Licomont BS 100 also function with the same mechanism of a viscosity reduction of the binder. Licomont BS 100 is a fatty acid amide which acts as a viscosity enhancer with a mechanism quite similar to that of Sasobit. Its physical properties are slightly different from that of Sasobit as it melts over the temperature range between 140 and 145 °C, and thus one requires production and compaction temperatures slightly higher than Sasobit-modified bitumen [11,29].

### 2.3. Asphaltan B

Asphaltan B, a blend of wax obtained by solvent extraction from lignite and fatty acid amides, is another commercially available wax with the mechanism to facilitate the production of WMA similar to that of Sasobit. The dynamics of the viscosity reduction in Asphaltan B-modified asphalt binder is similar to that of Sasobit-modified asphalt [30].

## 3. Chemical Additives

Chemical additives are some of the most recent emerging WMA technologies. They contribute to improving the ability of the asphalt binder to coat the aggregate particles rather than reducing the viscosity of the binder [31]. Chemical additives have a more diverse range of mechanisms through which they exert their function compared to other categories.

Chemical additives exist in the form of emulsions and surfactants, which work at the microscopic interface of the binder and aggregates to regulate and reduce the frictional forces at that interface within a range of temperatures (typically between 85 and 140 °C). The reduction and regulation of the frictional forces facilitate lubrication between the binder and aggregate during mixing and compaction, and this accounts for the improvement in adhesion obtained after the addition of chemical additives.

In the same way as waxes, some chemical additives (generally surfactants such as Rediset and Cecabase) reduce the binder viscosity while the emulsifying chemical additives (like Evotherm) improve lubrication in the aggregate/binder interface by altering some other parameters such as the surface free energy of the mix [32,33] thanks to their amphiphilic (surfactant) nature. This improvement in lubrication accounts for the asphalt mix particles moving over each other more easily which in turn lowers the mixing and compaction energy levels at lower temperatures [11].

It can be expected that another mechanism is concurrently present, i.e., the competitive interactions between the amphiphilic additive and the amphiphilic resins pre-existent in bitumen. In fact, it has been recently highlighted that, in addition to polar and apolar interactions, further specific interactions between surfactants themselves can trigger peculiar self-assembly processes [34,35] dictating the final overall aggregation pattern and the peculiar dynamics and transport processes [36,37]. There are several approaches that can be used to evaluate the durability of asphalt pavements and the moisture-induced damage potential of WMA. Two of the most viable and mechanistic approaches are the Surface Free Energy (SFE) approach and the Contact angle approach. Arabani et al. [38] reported a strong correlation between the moisture-induced damage potential of WMA mixes based on the SFE of asphalt mixes. Bhasin et al. [39,40] also suggested different combinations of SFE parameters such as the Work of Adhesion, Work of Debonding and Work of Cohesion also known as Cohesion Energy of aggregates to describe the moisture susceptibility of an asphalt binder–aggregate system as a single value.

The SFE of a material is the work required to create a unit area of a new surface in a vacuum. Hence, a high value is desirable for the durability and effectiveness of paved asphalt [41]. The total SFE (γ_total_) comprises three components, γ^LW^, γ^+^ and γ^−^, respectively, the non-polar Lifshitz van der Waals (LW), Lewis acid and Lewis base components. Depending on the state of the material, these components combined in different ratios produce the γ_total_ of the material [42]. The variability of these components defines the SFE parameters such as cohesion energy, work of adhesion, work of debonding and energy ratio. These parameters define the variable combination of the SFE of asphalt binder with that of the aggregates to give a durable pavement.

Previous research [41] has shown that the elemental composition (Carbon, Nitrogen, Hydrogen and Sulphur) of an asphalt binder is correlated to SFE parameters. Since additives in general alter the structure and physical properties of binders, it is necessary to study the effect that above all the chemical additives exert on the SFE parameters of both the binder and the aggregates.

The use of chemical additives has been proven to alter several parameters and their components, such as SFE, its components and contact angle parameters e [41,43], which will all be discussed later in this paper. The two most important SFE parameters which are altered by the application of chemical additives are work of adhesion and work of debonding. The former is defined as the work required to detach asphalt binder coating from aggregate surface in a dry state in their interface in vacuum [42]. It is also known as the *dry adhesion energy* and it is a parameter frequently measured to determine the effectiveness of warm mix. A high work of adhesion value implies a strong bond between the components of the warm mix, leading to a more durable and less moisture-susceptible warm mix. The work of debonding, also known as the *wet adhesion energy*, is another important parameter linked the reduction of system energy when the binder separates from the aggregate in the presence of water in a phenomenon called *stripping*. A high value of this parameter implies a higher thermodynamic potential for stripping to occur in the presence of water, hence a low value of this parameter is desired [43]. The γ_total_, the work of adhesion and the work of debonding are the most important energy parameters used to evaluate the durability and stability of warm mix asphalt. Several studies [41,43] have shown that the use of chemical additives in WMA have resulted in higher SFE values, higher work of adhesion values and lower work of debonding values.

### 3.1. Evotherm

One of the most commonly researched and used chemical additives is Evotherm^®^. Hurley and Prowell [44] demonstrated that, at a given compaction temperature, the addition of Evotherm to asphalt binder increases the resilient modulus of an asphalt mix compared to control mixtures having the same performance-graded (PG) binder. The first generation of Evotherm^®^ technology is a high residue emulsion known as Evotherm ET (Emulsion Technology). MeadWestvaco then introduced a second generation Evotherm technology where the chemical additives are injected as a solution into the asphalt line at the plant known as Evotherm DAT (Dispersed Additive Technology).

The latest and most recently emerging Evotherm Technology known as Evotherm 3G (3rd Generation) is a water-free WMA technology which allows the additive to be mixed with the binder at a terminal [15]. Evotherm 3G and Evotherm DAT have made Evotherm ET obsolete due to the convenience with which they can be incorporated into WMA. Evotherm ET is a binder-rich, water-based emulsion that contains about 70% asphalt binder. The water in the emulsion turns into steam when mixed with hot aggregates, thus facilitating better mixing and compaction.

The emulsifiers in the Evotherm^®^ are adsorbed onto the aggregate surface with a long hydrocarbon tail extending beyond the aggregate surface, which promotes interfacial adhesion between binder-aggregate interface surfaces [11,45]. It is highly possible that in Evotherm the long hydrocarbon tails of the emulsifiers extending beyond the aggregate surface, are responsible for the higher SFE values of the binder–aggregates interface.

The pH compatibility of binder and aggregates is also an important factor that influences the level of bonding in the warm mix. Since most asphalt binders are more acidic than basic [43], it is highly recommended to be careful using acidic aggregates such as granite with an asphalt binder which is also acidic in nature, as this may result in a weak bond between asphalt binder and aggregate, which will result in high value of work of debonding and, consequently, a higher susceptibility of the mix to moisture-induced damage [46]. It must be pointed out that the pH and the presence of acidic or basic species is a delicate matter, since they can greatly influence the intermolecular aggregation pattern giving sometimes unexpected structural and dynamic properties [47]. Several previous studies [43,48,49] have proven that the incorporation of Evotherm into WMA resulted in higher asphalt–aggregate interfacial SFE values whether applied to the binder or into the mix at the plant.

Ghabchi et al. [43] estimated γ_total_, work of adhesion, work of debonding and energy ratios to assess the moisture-induced damage potential of combinations of neat and Evotherm^®^ asphalt binders and different aggregates. They also measured and compared contact angle values of Evotherm-modified asphalt binder and unmodified neat asphalt binder.

Their results (reported in Table 2) indicated that the addition of 0.5% and 0.7% Evotherm^®^ resulted in an increase in both γ_total_ and work of adhesion and a reduction in work of debonding, implying a better aggregate–binder bond and thus lower moisture susceptibility potential. Their results also showed that the use of Evotherm^®^ resulted in reduced contact angles compared to those of unmodified asphalt binder. Yu et al. [50] carried out a study in which they combined two generations of Evotherm technology (Evotherm DAT and Evotherm 3G) to evaluate the WMA effect on the mechanical resistance of the binder. In the base sample, Evotherm DAT and Evotherm 3G were added in the proportions of 5% and 0.5% of the total weight, respectively. The results of the study showed improved fatigue resistance, moisture damage resistance and better workability.

In general, when the contact angle value of the binder and aggregate sample is ≥90°, it indicates low wettability, and the binder is unable to wet and coat the surface of the aggregate. When contact angles are <90°, there is some adhesion and the binder is able to wet the surface of the aggregate. For contact angle values ≈ 0°, spreading of the binder around the surface of the aggregate can occur and there is a strong adhesion. This parameter is what translates to the extent of coating of the aggregate by the asphalt binder. The implications of variations in contact angle on the properties of asphalt binder are expected to influence the SFE components and energy parameters such as moisture susceptibility potential and work of adhesion and debonding [41,43].

In addition to the effect of chemical additives, anti-stripping agents are also incorporated into WMA to improve its durability. They decrease the moisture susceptibility of warm mix asphalt by reducing the potential of moisture to disrupt the adhesive bond between binder and aggregate. Hydrated lime is a model anti-stripping agent and it has been proven to be highly effective in strengthening the adhesion between the asphalt binder and aggregates [51]. Some researchers attribute the increase in adhesive strength to changes in the surface chemistry or molecular polarity of the aggregate surface. This consequently leads to a stronger bond at the binder–aggregate interface [52,53]. Since hydrated lime is basic in nature, it is believed that its application in WMA increases the base component of the SFE of aggregates thus increasing the overall SFE value. These changes in aggregate SFE components lead to a significant improvement in adhesion between an asphalt binder and acidic aggregate that is more sensitive to moisture damage. The use of hydrated lime has also been proven to decrease aggregate polarity, thus decreasing the affinity of aggregate surface, which has polar molecules, to water [52]. Apart from the treatment of WMA with anti-stripping agents, the type of stones used as aggregate has to be carefully chosen. This is because different stones have different polarities and SFE parameters which leads to a variability in adhesion potential when incorporated into the warm mix. Hesami et al. [52] proved that the free energy of adhesion between water and granite aggregate is much greater than that between water and limestone aggregate, indicating that the affinity of granite to water is higher compared to limestone.

### 3.2. Rediset

Rediset is another chemical additive produced by Akzo Nobel that contains cationic surfactants and rheology modifiers (of organic nature). It is a polyfunctional additive based on fatty amine surfactants and olyethylenes [54]. This additive contains a long chain aliphatic hydrocarbon structure and an ${-\mathrm{NH}}_{3}^{+}$ group, which reacts chemically with aggregate surfaces [55]. Rediset has a slightly different mechanism compared to other chemical additives because it contains in-built anti-stripping agents, which reduce susceptibility to moisture damage [56]. Rediset is regarded as a polyfunctional additive because it functions by reducing the interfacial friction between thin films of the asphalt binder and the coated aggregates, while also improving workability by increasing lubrication and allowing mixing and compaction at reduced temperatures [11]. The surfactant part of this product (similar to chemical additives) decreases the surface tension of asphalt binder and improves the wettability of the aggregate by using an asphalt binder [56]. The organic part reduces the viscosity of the asphalt binder and provides a lubricating effect for easier coating and compaction [32]. It has also been reported that Rediset positively changes kinematic and dynamic viscosity as well. Studies in the literature [57,58] showed that Rediset decreases the kinematic viscosity of asphalt binder at 135 °C. Van de Ven et al. [59] also demonstrated that Rediset has a strong effect on the dynamic viscosity at 110 °C for a hard asphalt binder using Dynamic Shear Rheology results with a cone and plate device. The use of Rediset has also been shown to have similar effects on the SFE parameters, as was observed with the use of Evotherm [60]. Cecabase RT is another chemical additive produced by CECA (France), which has the same hypothesized mechanism to produce WMA as that of surfactants such as Rediset [11].

### 3.3. Iterlow

Iterlow is a liquid chemical additive produced by Iterchimica (Italy) which, when added to the asphalt binder, allows for the production of WMA at temperatures above 120 °C. According to Hill et al. [61], liquid chemical additives generally act as emulsifying agents and contain amine groups that can improve the cracking resistance at low service temperatures and the resistance to moisture damage. Just like other chemical additives, Iterlow improves the workability of the mix and facilitates paving and mixing at lower temperatures (between 90 °C and 120 °C) depending on the type of bitumen. Iterlow has little or no effect on the bitumen grade [60].

## 4. Foaming Technologies

Foaming technologies involve the introduction of small amounts of water which are delivered into the binder using different methods. Although the method of water delivery is different, foaming processes generally conform to one underlying concept, which is the expansion factor of water after transition from liquid to vapor state. The expansivity of water by a factor of about 1700 when it is converted into steam is the mechanism behind the effectiveness of foaming technologies in general [62]. The latent steam in the form of foam causes an overall reduction in the viscosity of the asphalt binder which is facilitated by an increase in volume and surface area of the binder and this results in improved aggregate coating and easier compaction of the asphalt mix at lower temperatures [11]. As mentioned earlier in this review, foaming technologies are divided into two processes, namely, *water-bearing* and *water-based* processes. Water-bearing processes involve the use of water-containing technologies which combine water foaming and additive (zeolites) dosage. These processes involve the incorporation of hydro-thermally crystalized minerals called zeolites into asphalt binder. Upon contact of the zeolite with hot binder, water is released from the zeolite’s crystal structure without changing the volume and structure of the crystal. The released water changes to water vapor and causes foaming. There are two groups of zeolites namely; synthetic zeolites which are produced from chemical reactions, and natural zeolites which are formed by naturally occurring geological processes [63,64,65,66]. Water-based processes, on the other hand, do not actually involve the use of additives but involve the direct injection of water into a binder, which generates microscopic bubbles and thus causes foaming in the binder [11,67]. Water-based processes involve the injection of pressurized cold water into hot asphalt using specially designed injection nozzles. These processes do not chemically modify the asphalt binder during the production of foam resulting from the addition of water. The foaming only occurs in order to enable for easier aggregate coating. Water-based processes eliminate the need for expensive additives due to its general technique of directly injecting small amounts of water (generally with a mass ratio of between 1% and 5% to the mass of binder) into the hot binder to form microscopic bubbles (which creates the foam) in the continuous phase. These processes rely solely on the foaming action of steam when water is injected into hot asphalt. WAM Foam (Warm Asphalt Mix Foam) and Double barrel green are examples of water-based processes [11]. Unlike organic additive techniques, the reduction in viscosity of binder caused by foaming processes is temporary and only for a short time usually during mixing and compaction of the asphalt mix after which the foam collapses and the asphalt binder reverts to its normal binder state. This technology reduces WMA production temperatures by between 20 and 30 °C.

### Water-Bearing Processes

The main foaming technologies that concern this review are the water-bearing processes. These processes involve the use of porous, hydrated aluminosilicate minerals called zeolites. Zeolites have a general formula of M_*x*/*m*_[(AlO_2_)_*x*_(SiO_2*y*_)]. H_2_O, where the M_*x*/*m*_ unit constitutes ion-exchangeable cations, and the [(AlO_2_)*_x_*(SiO_2*y*_)] unit is the zeolite crystalline framework. The apices of the SiO_4_ and AlO_4_ tetrahedrons are connected by oxygen atoms creating a three-dimensional spatial network in which voids are formed in the form channels and chambers. This defines the crystallinity of these aluminosilicates. The Si/Al ratio in the crystalline framework determines factors such as the mineralogical composition of the zeolite, size of channels and chambers, ion-exchangeable capabilities and hydrophilic–hydrophobic properties [64]. In addition to these features, water molecules are bound to the zeolite crystals and are released when subjected to high temperatures without changing the zeolite structure. Zeolites are either naturally occurring or synthetic although the synthetic zeolites are the most commonly used group of zeolites for WMA. Natural zeolites are microporous, hydrated aluminosilicates, which are generally used as commercial adsorbents [67,68]. The most common, naturally occurring zeolites are Clinoptilolite and Phillipsite. Clinoptilolite is a common natural zeolite and comprises of microporous tetrahedral arrangements of silica and alumina [69]. It is used in the production of cement, concrete and asphalt due to its large distribution of micropore spaces and high resistance to extreme temperatures [70].

Synthetic zeolites are finely powdered hydrated sodium aluminosilicates, which have been usually hydro-thermally crystallized. They have a complex structure, usually with a porous morphology [71] and sometimes with fractal arrangement conferring high surface-to-volume ratio [72]. Common examples of synthetic zeolites are sodium silicates (Na_2_SiO_3_) and sodium aluminates (NaAlO_2_) each of which are categorized into types. The most common types are ZSM-5, X, Y, A, and NaP1 types [65,66]. Synthetic zeolite technologies such as Aspha-min and Advera belong to the same group of Linde A (LTA) structure-type synthetic zeolites and are the most commonly used zeolites for WMA. Advera^®^ which is an additive produced by PQ Corporation is a new generation of the Aspha-min technology. These additives contain between 18% and 22% of water by mass which is released at higher temperatures facilitated by the mixing and compaction processes [11,32,73]. The recommended dosage of Advera in the Warm Mix is 0.25% by weight of the mix. During the mixing process, due to the elevated temperature, the water contained in the zeolite is released into the binder and foaming occurs. As a result of this, a reduction of binder viscosity and increase in workability are observed. Previous research studies have suggested that water released from Advera condenses and is reabsorbed by the zeolite which reduces moisture susceptibility [73,74,75]. This in-built anti-stripping feature of Advera makes it more commonly used in the asphalt industry because foaming technologies in general must introduce enough water into the binder to cause foaming without adding so much to induce stripping. Aspha-min works in a mechanism very similar to Advera. Aspha-min is a sodium aluminosilicate produced by Eurovia GmbH, (Germany) who recommend that the dosage of Aspha-min should be 0.3% by weight of mix. This additive releases water at a temperature range of 85–180 °C. During the mixing process, both the additive and binder are added simultaneously to the aggregates. The water trapped inside the additive is released and this expands the volume of the binder while foaming occurs. This facilitates aggregate coating of the mixture at lower temperatures [76,77].

Advera and Aspha-min have similar effects on WMA due to the similarities in their chemical compositions and mechanism of action. The only slight difference is the anti-stripping characteristic that Advera possesses. This feature is absent in Aspha-min, thus anti-stripping agents are recommended to be used in combination with Aspha-min [78]. In the National Center for Asphalt Technology (NCAT) report [76], it was pointed out that the moisture susceptibility and stripping potential were decreased upon addition of an anti-stripping agent (1.5% hydrated lime) to the foamed mix. Another notable observation about WMA foaming processes is that, since the introduction of water into the binder only brings about a temporary reduction in viscosity, it is highly preferable to introduce water into the binder in steps. This stepwise addition of water ensures consistent workability for longer periods. Barthel et al. [79] proved that a stepwise release of water creates a controlled foaming effect and prolongs the timeframe in which there is an improved workability of the mix. This facilitates better coating of aggregates by the binder. In water-based processes, the entire water content of the zeolite mineral is not released into the warm mix. This is due to the fact that zeolites can continuously release water while being subjected to temperatures as high as 400 °C. The lower processing temperature of Warm Mix Asphalt means that the zeolites still retain some of the water in their structures. The amount of released zeolite water at WMA production temperatures can be estimated by thermal analysis [80].

## 5. Super-Stabilized Emulsions and other WMA Techniques

**Figure 3:**
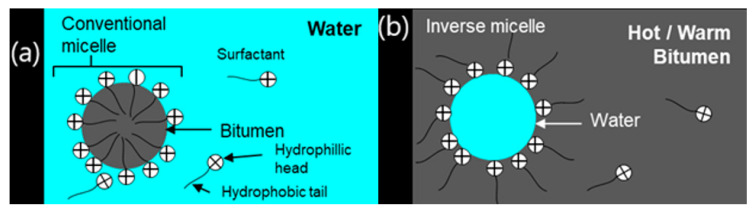
(**a**) Traditional bitumen emulsion. (**b**) Stabilized bitumen emulsion. Reprinted with permission from [81]. Copyright (2019), with permission from Elsevier.

Several other techniques exist for producing WMA, which involve methodologies slightly different from the generally accepted techniques. Hybrid WMA technologies such as Tri-Mix Warm Mix Injection System and Low Energy Asphalt, constitute some of these techniques. One less common technique for WMA production is the use of stabilized emulsion to produce Warm Mix. As mentioned earlier, this procedure is even more environmentally friendly and is also very cost effective because it does not really involve the use of additives in the form of products. Instead, it utilizes chemically stabilized bituminous emulsions systematically added into the mix to improve its quality and durability. In this process, the aggregates are first pre-coated with the emulsion before the binder is added to the mix, and sometimes the binder is foamed before being added to the mix, making it a hybrid technology [81]. The combination of these processes most likely increases workability of the mix because of the reduced viscosity of the binder and also the adhesion parameters like SFE of the aggregates, which have been favorably changed due to the pre-coating of aggregates by the stabilized emulsion. Since an emulsion consists of a dispersion of small droplets of one liquid in another liquid, stabilized emulsions have three important components: water, bitumen and an emulsifying agent (surfactant) which is chemically composed of large molecules and functions to reduce interfacial tension in the mix [14]. Chemically stabilized (non-traditional) bituminous emulsions have better characteristics in WMA compared to traditional bituminous emulsions. Prowell [82] found it worthy to note that non-traditional bitumen emulsions have been developed for the production of WMA. The main advantage of stabilized emulsion over traditional emulsion is that, upon mixing, most of the water evaporates and surfactants converge to form inverse groups of molecules or micelles (shown in Figure 3b), which is not the case with traditional emulsion (shown in Figure 3a).

The inverse micelles prevent evaporation of the remaining water and forms a thin film of water between the aggregates, improving the workability and compaction of the mixture. Once the aggregate and bitumen have been mixed and compacted, the remaining water evaporates by chemical reactions that are designed during emulsion formulation [81,83]. Chemical additives which are emulsion-based exist and are also used for WMA production, although these products cost more. An example of a super-stabilized emulsion is Evotherm^®^ and has been described in Section 3.1. These are emulsions that have been stabilized and additive technology has been added to the formulation. These emulsion-based products are called *super-stabilized emulsions*. This stability is achieved by a special formulation of the liquid phase containing additives, which impart higher stability to the emulsion [81]. The combination of these stabilized emulsions with surfactants or polymers can improve mixture workability, coating properties and result in more effective compaction at lower temperatures [84].

## 6. Conclusions

Increasing concern regarding environmental issues is the most stimulating factor behind the drift of the asphalt industry towards Warm Mix Asphalt (WMA). WMA is a new, fast emerging technology and the validation of facts about this technology will contribute to its widespread acceptance. The lack of a standardized WMA mix design increases the need for an understanding of the underlying mechanisms of action of additives in the mix. Lack of knowledge on the potential and dynamics of WMA in several parts of the world where the technology discoveries are not widespread is a limiting factor on the growth of this technology. This is partly due to the limited knowledge on WMA additives which does not encourage proficiency with WMA techniques on the part of industry personnel. The discussions presented in this paper, fostered by a review of previous research and studies, highlight the following points about WMA additives and technology:

1. Organic additives in the form of waxes and fatty amides act as flow modifiers by melting below the melting point of the binder, thus reducing its viscosity during mixing which improves the coating and workability of the mix.
2. During the cooling phase of the mix, waxes such as Sasobit start to crystallize and form a microscopic lattice structure in the binder which results in the increased stiffness of the asphalt pavement. This is responsible for the deformation resistance and reduction in the amount of air voids observed in wax-treated WMA. It is speculated that the stiffness observed in the improved mix results from the alteration of binder hydrocarbon chain length by organic additives which are hydrocarbon-rich in nature.
3. Chemical additives in the form of emulsions and surfactants function at the microscopic interface of the binder and aggregates to regulate and reduce the frictional forces at that interface. This improves lubrication between the binder and aggregates.
4. Emulsifying agents such as Evotherm generally improve lubrication of the mix by altering Surface Free Energy components and parameters. This is responsible for asphalt mix particles moving more easily over each other in the mix which in turn translates to better coating of aggregates caused by an improved contact angle. Liquid chemical additives like Iterlow also act as emulsifying agents which contain amine groups and improves cracking resistance of the mix at low temperatures.
5. Surfactants such as Rediset generally reduce surface tension of asphalt binder to improve wettability of aggregates. They also function in a similar fashion as the organic additives by reducing the viscosity of the binder to improve workability of the mix.
6. Foaming technologies in the form of Zeolites (aluminosilicates) and water-based processes (injection nozzles) generally reduce binder viscosity temporarily. This improves coating and mix workability. These processes are more susceptible to moisture damage due to the involvement of water in the foaming process thus anti-stripping agents are often added to the mix if it is not already contained in the product.
7. Hybrid techniques such as Sasoflex and Tri-Mix Warm Mix Injection system combine different categories of additives with specific desirable features to synthesize additives with versatile functions. This might be the future of WMA technology.

Each category of additive is characterized by specific mechanism(s) of action in Warm Mix Asphalt. Some additives combine several mechanisms such as conjoining improved lubrication with reduced viscosity while altering several adhesion and cohesion parameters of the mix. The ability of additives to exploit several mechanisms to improve Warm Mix is a characteristic that can be better exploited in the asphalt industry to produce high-performance WMA. The establishment of facts regarding mechanism of action of additives is key to formulating a standard design mix procedure and the advancement of asphalt production technologies in general.

## Figures and Tables

**Table 1:** Products used in Warm Mix technology. Reprinted with permission from [9]. Copyright (2011), with permission from Elsevier.

WMA Processes	Product	Company	Description	Dosage of Additive	Country Where Technology Is Used	Production Temp. (or Reduction Range) °C
***Organic additives***						
FT Wax	Sasobit^®^	Sasol	Fischer-Tropsch Wax	1.0–2.5% by weight of binder	Worldwide	(20–30 °C)
Montan Wax	Asphaltan B	Romonta GmbH	Montan Wax with fatty acid amide	2.0–4.0% by mass of bitumen	Germany	(20–30 °C)
Fatty Acid Amides	Licomont BS	Clariant	Fatty acid amide	3.0% by mass of bitumen	Germany	(20–30 °C)
Wax	3E LT or Ecoflex	Colas	Proprietary	Not specified	France	(20–30 °C)
***Chemical additives***						
Emulsion	Evotherm^®^ technologies	MeadWestvaco	Chemical packages with or without water	0.5–0.7% by mass of bitumen	USA, worldwide	85–115 °C
Surfactant	Rediset	Akzo Nobel	Cationic surfactants & organic additive	1.5–2.0% by weight of bitumen	USA, Norway	(30 °C)
Surfactant	Cecabase RT	CECA	Chemical package	0.2–0.4% by mixture weight	USA, Norway	(30 °C)
Liquid Chemical	Iterlow	IterChimica		0.3–0.5% by mass of bitumen	Italy	120 °C
***Foaming Processes***						
Water-containing	Aspha-Min^®^	Eurovia and MHI	Water-containing technology using zeolites	0.3% by total weight of mix	Worldwide	(20–30 °C)
Water-containing	Advera^®^	PQ Corp.	Water-containing technology using zeolites	0.25% by total weight of mix	USA	(10–30 °C)
Water-based	WAM Foam	Shell and Kolo-Veidekke	Foamed binder	2–5% water by mass of binder	Worldwide	100–200 °C

**Table 2:** SFE components of PG64-22 asphalt binder modified with Evotherm^®^ and aggregates (Reprinted with permission from [43]. Copyright (2013), with permission from Elsevier). γ^LW^: LW component. γ^−^: Lewis base component. γ^+^: Lewis acid component. $\gamma^{+-}=2\sqrt{\gamma^{+}\gamma^{-}}$: acid–base component. γ_total_: total surface free energy of the material.

Material Type	Additive (%)	Surface Free Energy Components (mJ/m^2^)					
		γ^LW^	γ^−^	γ^+^	γ^+−^	γ_total_	γ^+^/γ^−^
*PG64-22 Binder with different % additive*							
Neat	0%	9.44	0.93	1.22	2.13	11.57	1.30
Evotherm^®^	0.25%	6.84	1.24	3.45	4.14	10.99	2.77
	0.50%	6.74	2.50	3.03	5.50	12.24	1.21
	0.75%	9.17	3.03	5.50	4.52	13.69	1.82
*Aggregates from Testing and Literature*							
Limestone (Tested)	-	51.4	741.4	17.5	227.8	279.2	0.024
Granite	-	133.2	96	24.1	96.2	229.4	0.251
Basalt	-	52.3	164	0.6	19.8	72.1	0.004
